# The association between schizophrenia and white blood cells count: a bidirectional two-sample Mendelian randomization study

**DOI:** 10.1186/s12888-023-04760-6

**Published:** 2023-04-19

**Authors:** Zibo Gao, Biao Li, Xinru Guo, Wei Bai, Changgui Kou

**Affiliations:** 1 Department of Epidemiology and Biostatistics, School of Public Health, Jilin University, 1163 Xinmin Street, Changchun, Jilin Province 130021 China; 2 Unit of Psychiatry, Department of Public Health and Medicinal Administration, & Institute of Translational Medicine, Faculty of Health Sciences, University of Macau, Avenida da Universidade, Taipa, Macau, SAR 999078 China

**Keywords:** White blood cells, Schizophrenia, Mendelian randomization

## Abstract

**Background:**

Positive associations between the risk of schizophrenia and the level of white blood cells (WBC) count have been suggested by observational studies. However, the causality of this association is still unclear.

**Methods:**

We used a group of bidirectional two-sample Mendelian randomization (MR) analyses to estimate the causal relationship between schizophrenia and WBC count traits (i.e., WBC count, lymphocyte count, neutrophil count, basophil count, eosinophil count, and monocyte count). The threshold of FDR-adjusted *P* < 0.05 was considered as showing potential evidence of a causal effect. Instrument variables were included based on the genome-wide significance threshold (*P* < 5 × 10^− 8^) and linkage disequilibrium (LD) clumping (r^2^ < 0.01). In total, 81, 95, 85, 87, 76, and 83 schizophrenia-related single nucleotide polymorphisms (SNPs) were used as genetic instruments from Psychiatric Genomics Consortium for six WBC count traits, respectively. And in reverse MR analysis, 458, 206, 408, 468, 473, and 390 variants extracted from six WBC count traits were utilized as genetic instruments, which were obtained from a recent large-scale Genome-Wide Association Study (GWAS).

**Results:**

Genetically predicted schizophrenia was positively associated with the level of WBC count [odds ratio (OR) 1.017, 95% confidence interval (CI) 1.008–1.026; *P* = 7.53 × 10^− 4^], basophil count (OR 1.014, 95%CI 1.005–1.022; *P* = 0.002), eosinophil count (OR 1.021, 95%CI 1.011–1.031; *P* = 2.77 × 10^− 4^), monocyte count (OR 1.018, 95%CI 1.009–1.027; *P* = 4.60 × 10^− 4^), lymphocyte count (OR 1.021, 95%CI 1.012–1.030; *P* = 4.51 × 10^− 5^), and neutrophil count (OR 1.013, 95%CI 1.005–1.022; *P* = 0.004). WBC count traits are not associated with the risk of schizophrenia in our reverse MR results.

**Conclusion:**

Schizophrenia is associated with elevated levels of WBC count (i.e., higher WBC count, lymphocyte count, neutrophil count, basophil count, eosinophil count, and monocyte count).

**Supplementary Information:**

The online version contains supplementary material available at 10.1186/s12888-023-04760-6.

## Background

Schizophrenia is a complex behavioral and cognitive syndrome, which is considered one of the most serious psychiatric disorders. It causes a significant global disease burden and heavy economic burden with a suicide rate of around 5% [1]. Many genetic epidemiological studies have shown that schizophrenia is highly polygenic heritability with the rate ranging from 64 to 81% [2–4].

In recent years, the relationship between psychiatric disorders and the inflammatory response has been explored in many studies [5–7]. For example, a large cohort study showed that both C-reactive protein (CRP) and leukocytes were significantly associated with both negative and positive symptoms in patients with psychiatric disorders [8]. Numerous avenues of inquiry have suggested the relationship between psychiatric disorders and immune dysfunction (e.g., aberrations in immune cells number and inflammatory markers) [9–11]. White blood cells (WBC) are the hallmark cells of the body’s inflammatory response. Several cross-sectional studies and meta-analyses have already suggested that there are increased total and differential WBC counts in patients with schizophrenia, and immune cells might have a significant impact on schizophrenia [12–14]. It should be noted that previous studies usually relied on observational studies, which may suffer from confounding factors like reverse causality and selection bias, and meta-analyses have also reported substantial heterogeneity. At the same time, current studies have shown that various antipsychotics have a significant effect on the immune cells count of the body [15, 16]. Therefore, it remains unknown if the alterations of the immune cells number in schizophrenia are causal or potentially compensatory reactions to upstream dysfunction.

Mendelian randomization (MR), a more powerful method for causal inference than the traditional analysis approach (e.g., case-control study) used in observational studies, uses genetic variations as instruments to infer causality, thus overcoming the potential problems of reverse causality and confounding in observational studies [17, 18]. In recent years, thousands of genetic variant loci associated with complex traits have been identified by GWAS, which allowed MR to be widely used in causal inference studies of mental disorders [4, 19–21]. Recent studies have used MR analyses to explore the potential causal relationship between inflammatory biomarkers levels and partial blood cell trait with schizophrenia [22–24], but the sample size of those studies was not large enough compared with the summary data from new GWAS meta-analysis, and the categories of WBC traits are incomplete. Here, we investigate the causality between schizophrenia susceptibility and WBC count traits via a two-sample bidirectional MR method comprehensively.

## Methods

### Study design

**Fig. 1:**
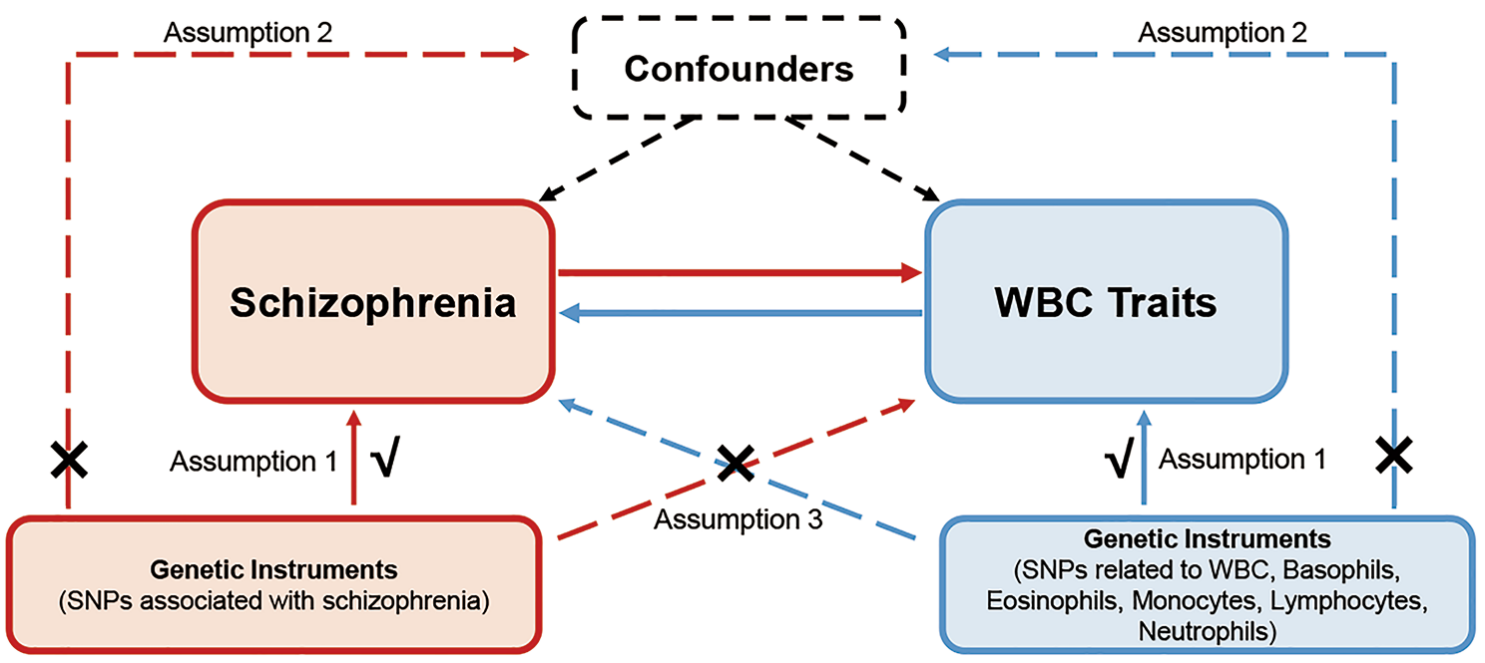
Schematic representation of this bidirectional MR study. WBC, white blood cells; SNPs, single nucleotide polymorphisms

The relationship between schizophrenia and WBC count traits was investigated using 12 MR analyses (Fig. 1). There are three key assumptions that underlie MR. (1) a significant association exists between genetic variants and exposure; (2) there is no correlation between the instrument variables (IVs) and any confounding factors; (3) the exposure is the only way in which genetic variants affect the outcome of interest [25]. We adopted the GWAS summary statistics for schizophrenia and six count traits of WBC (i.e., WBC, lymphocyte, neutrophil, basophil, eosinophil, and monocyte). The six WBC count traits in this study are all the total absolute count. A forward MR analysis was performed to test the causal effect of genetically predicted schizophrenia on WBC count traits. And the reverse analysis was utilized to examine the effects of WBC count traits on schizophrenia risk. Most of the participants in our analyses were of European ancestry. Ethical approval can be obtained from all original studies.

### Selection of instrumental variants

The summary statistics of 6 peripheral blood WBC count traits were derived from a meta-analysis of 408,112 participants [26]. Summarized data for schizophrenia were obtained from the Psychiatric Genomics Consortium (PGC) [4]. The detailed sources of the data are shown in Supplementary Table 1. The single nucleotide polymorphisms (SNPs) were retrieved from GWAS summary statistics mentioned above as IVs, which were included based on the genome-wide significance threshold (i.e., *P* < 5 × 10^− 8^) and linkage disequilibrium (LD) clumping (r^2^ < 0.01).

### Statistical analyses

In this investigation, *F*-statistic was calculated as

1 $$F=\left(\frac{n-k-1}{k}\right)\left(\frac{{R}^{2}}{1-{R}^{2}}\right)$$

and *F* ≥ 10 was typically recommended for strong instruments [27]. The *R*^*2*^ value was calculated to determine how much variance IVs are explained in the exposure factor. We used the inverse-variance-weighted (IVW) random-effects model to generate effect estimates as the major result, and the multiplicative random effect model would be adopted if there was evidence of heterogeneity. The MR-Egger regression and weighted median were used for alternative analyses. The MR-Egger intercept was used to determine directional horizontal pleiotropy. And then the MR-PRESSO test was used to remove detected outliers to correct for horizontal pleiotropy [28]. The heterogeneity between individual SNPs was tested by Cochran’s Q statistics and funnel plots. And the leave-one-out sensitivity analysis was performed to test whether the main results were driven by any individual SNP. The Benjamini-Hochberg false discovery rates (FDR) correction was used to adjust *P* values in MR analyses. The threshold of FDR-adjusted *P* < 0.05 was considered as showing potential evidence of a causal effect and all *P* values are two-tailed [29]. MR analyses mentioned above were conducted by using the MRInstruments, TwoSampleMR, and MRPRESSO packages with R (version 4.1.2).

## Results

### Forward MR analyses of the effects of schizophrenia on WBC count traits

**Fig. 2:**
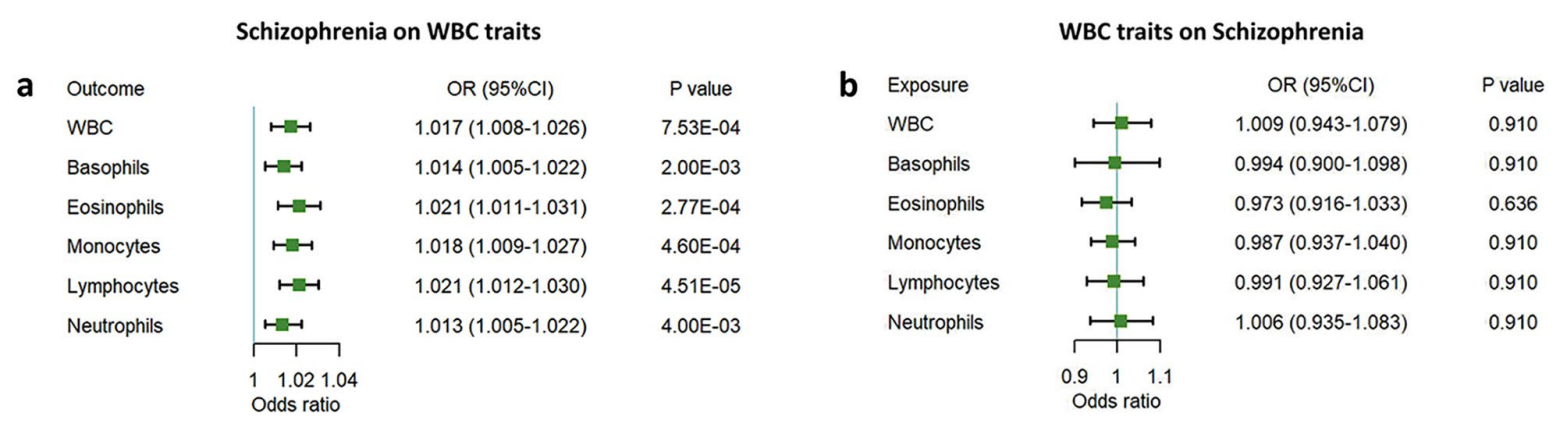
MR estimates of the effects between schizophrenia and WBC count traits. a, forward MR analysis; b, reverse MR analysis; WBC, white blood cells count. Odds ratios and 95% confidence intervals were derived using the inverse-variance weighted random-effects model.

Firstly, the causal effects of schizophrenia on 6 WBC count traits were investigated. After removing outliners due to horizontal pleiotropic and ambiguous palindrome, we finally included 81, 76, 83, 95, 85, and 87 variants as genetic instruments for WBC count, lymphocyte count, neutrophil count, basophil count, eosinophil count, monocyte count in the forward MR analyses, respectively (Supplementary Tables 5–10). Figure 2a and Supplementary Table 3 present the forward MR results; Supplementary Figs. 1–4 present the scatter plots, forest plots, funnel plots, and leave-one-out analysis results, respectively. Within the six one-way analyses, the smallest *F*-statistic was equal to 28.86, showing a weak instrumental bias, which was unlikely to have an impact on the IVW estimates (Supplementary Table 2).

The association between schizophrenia and the level of WBCs count in forward MR analyses were all statistically significant via the IVW model [odds ratio (OR) 1.017, 95% confidence interval (CI) 1.008–1.026; *P* = 7.53 × 10^− 4^]. The same result arises in the weighted median model (OR 1.015; 95%CI 1.006–1.025; *P* = 0.009). As to five WBC count traits, in the IVW models, genetically predicted schizophrenia was positively associated with levels of basophil count (OR 1.014, 95%CI 1.005–1.022; *P* = 0.002), eosinophil count (OR 1.021, 95%CI 1.011–1.031; *P* = 2.77 × 10^− 4^), monocyte count (OR 1.018, 95%CI 1.009–1.027; *P* = 4.60 × 10^− 4^), lymphocyte count (OR 1.021, 95%CI 1.012–1.030; *P* = 4.51 × 10^− 5^), and neutrophil count (OR 1.013, 95%CI 1.005–1.022; *P* = 0.004) (Supplementary Table 3). The MR-Egger intercepts indicated that no evidence of horizontal pleiotropy was found (all *P*-Egger intercepts > 0.10). Cochran’s Q statistics suggested the existence of heterogeneity (all *P*-Q values < 0.001) so the multiplicative random effect model was adopted. But there is no evidence of heterogeneity as shown in the funnel plot (Supplementary Fig. 3). Besides, the results of the leave-one-out analysis suggested the robustness of the observed causality.

### Reverse MR analyses of the effects of WBC count traits on schizophrenia

After removing the unavailable and the palindromic variants, we utilized 458, 473, 390, 206, 408, and 468 variants for WBC count, lymphocyte count, neutrophil count, basophil count, eosinophil count, and monocyte count as genetic instruments, respectively, where weak instrumental bias have been ruled out (Supplementary Tables 2, Supplementary Tables 11–16). The results of MR analysis by using MR Egger, weighted median, and IVW method all indicated that neither WBC nor the other five traits were causally related to schizophrenia (Fig. 2b and Supplementary Table 4). There was no horizontal pleiotropy in the reverse analyses (all MR-Egger intercept *P* values > 0.1). And the funnel plots (Supplementary Fig. 6) suggested no significant asymmetry of the MR analyses. However, Cochran’s Q statistics indicated the existence of heterogeneity (Q ≥ 357.50, all *P* values < 0.001).

## Discussion

To the best of our knowledge, this is the first bidirectional two-sample MR research that, examines the causal effects of six WBC characteristics in schizophrenia susceptibility. This MR study indicated that schizophrenia was positively associated with levels of WBC, lymphocyte, neutrophil, basophil, eosinophil, and monocyte. No evidence in the reverse MR analyses was observed that six WBC count traits were associated with schizophrenia.

Leukocytes are an essential component of the body’s immunological defense mechanism. In total, there are five types of leukocytes in the blood, namely neutrophils, eosinophils, basophils, lymphocytes, and monocytes, each of which has a specific physiological function. A recent meta-analysis showed that peripheral blood WBC count, neutrophil count, and monocyte count were significantly elevated in both patients with first-episode of psychosis (FEP) and chronic psychosis [30], which is consistent with the results of this study. However, possibly limited by heterogeneity between studies and small sample size, no correlation was found between several other categorical counts and schizophrenia in this meta-analysis. Newly published findings showed consistent results of elevated WBC and neutrophil counts in drug-naive FEP patients [31]. Several studies have pointed out that impaired inflammatory regulation represented by elevated WBC may be a manifestation of more severe neuropathological changes and associated with a higher risk of metabolic syndrome in patients with schizophrenia [31–33]. And it is worth noting that schizophrenia patients with increased WBC and neutrophil counts may show a tendency for increased self-aggression [34]. Neutrophils, a complex of transcriptionally active cells, have multiple essential roles such as secreting cytokines, regulating the activity of neighboring cells, and regulating macrophages for a long-term immune response [35]. As for studies on schizophrenia, in FEP patients, elevated neutrophil counts may serve as an important monitoring indicator and indirect evidence of reduced gray matter tissue, increased cerebrospinal fluid volume, higher PANSS scores, and more severe clinical presentation in patients [36]. The results of studies on lymphocyte count in patients with schizophrenia are not yet consistent. Some studies suggested that patients with schizophrenia exhibit a blood cell pattern of increased neutrophils, decreased lymphocytes, and increased neutrophil-to-lymphocyte ratio [37, 38]. However, there is evidence indicating that pro-inflammatory-prone monocytes were significantly overrepresented and the T-lymphocyte network was significantly activated in patients with recurrent-onset schizophrenia [14]. In addition, a study of brain tissue from patients with schizophrenia and mood disorder found that high-density lymphocyte infiltration may predict the onset of neuroinflammation associated with blood-brain barrier damage [39]. The correlation between eosinophil and basophil counts and schizophrenia was less well studied. No differences in eosinophil count between FEP patients and healthy controls have been observed [30]. However, some studies have indicated that eosinophil levels were reduced in patients with FEP and tended to rebound with the course of treatment [40].

Current research has demonstrated that psychiatric disorders are closely linked to inflammatory responses, and the inflammatory immune mechanisms of schizophrenia have received extensive attention. On the one hand, lots of observational studies suggested that patients with schizophrenia exhibit many characteristics of low-grade inflammation, for example, higher plasma levels of inflammatory cytokines, chemokines [31, 41, 42], and inducers [43]. Interestingly, however, several MR studies have successively reported that genetically predicted higher level of CRP may be a protective factor of schizophrenia [22, 44, 45]. On the other hand, studies have also suggested that higher levels of inflammatory mediators are associated with the pathogenesis and disease progression of psychiatric disorders [41, 46]. Cytokines may enter the brain tissue associated with neurological disorders to drive neuroinflammation and activate the neuroendocrine axis, thereby triggering depressive behavior and cognitive impairment [47–49]. It is interesting to note that we reviewed the results of previous GWAS studies and found that the loci significantly associated with schizophrenia and leukocyte count all contained SNPs associated with Y RNAs. And human Y RNAs have the primary function of mediating the initiation of chromosomal DNA replication and regulating the autoimmune protein Ro60 [50, 51]. In addition, previous studies have identified a common genetic link between lymphocyte count and schizophrenia in the MHC region [23]. And numerous studies have also identified the association between immunomodulatory genes and the increased risk of several psychiatric disorders, such as schizophrenia and major depressive disorder [4, 52]. All this evidence suggests that the underlying cause of elevated leukocytes in schizophrenia patients may be closely related to immune inflammatory responses and immune dysfunction. The results of the present study point to a significant unidirectional causal relationship between schizophrenia and WBC count traits, which may provide new evidence to support early inflammatory mechanisms in schizophrenic patients, suggesting the need for monitoring of WBC count traits and the management of low-grade inflammatory responses in SCZ patients. Besides, this study for the first time suggests that schizophrenia may be potentially causally related to elevated levels of eosinophils and basophils, and further studies are needed to verify the causal relationship found in this study.

The main advantage of the present study is that we used a bidirectional IVs analysis to avoid reverse causality and confounding factors as much as possible. To address the inability of cross-sectional studies to determine causality, Astle’s group carried out a series of one-way MR analyses in 2016 (n = 173,480), and only the causal relationship between lymphocyte count and schizophrenia was detected in the result [23]. Then, the potential causal association of neutrophils, and lymphocytes on susceptibility to three psychiatric disorders (seminal schizophrenia, major depression, bipolar disorder) was explored again in a bidirectional MR analysis published in 2021 using summary data published in 2016 [23], however, all the results were not significant, and vice versa [24]. In contrast, the results of the present study comprehensively showed a unidirectional positive correlation between schizophrenia and the levels of all six WBC count traits. This may be because the sample size of GWAS data used in this study was 2.3 times larger than the data used in the previous MR study [23, 24], and therefore this study had a higher power for causal association testing and more accurate effect estimates. Also, this study used different methods to estimate the causal effects, and the mutually corroborating results made the causal effects more convincing. However, several limitations cannot be ignored in this study. First, although the funnel plots all showed symmetrical distributions of IVs and the results of the leave-one-out analysis demonstrated the robustness of the results, Cochran’s Q values still showed the presence of heterogeneity. Unfortunately, subgroup analyses could not be performed for this study because neither primary data nor subgroup summary statistics data were available. Second, although this study showed no horizontal pleiotropy by the MR-Egger intercepts, some potentially pleiotropic IVs could not be completely excluded. What’s more, the practical effects of IVs on outcome variables cannot be fully explained yet. Finally, it is important to note, that the present evidence can only be limited to the European population because potential heterogeneity among different ethnic groups. Therefore, the present evidence can only be limited to the European population.

Therefore, we highlight that the results of this study should be interpreted cautiously and more studies are needed in the future to elucidate and validate the leukocyte count changes in schizophrenia and their inflammatory mechanisms.

## Conclusions

In conclusion, this bidirectional MR study indicated a positive causal effect of schizophrenia on the elevated levels of WBCs (including five white blood cells classification count traits). No causal effects of WBC count traits on the susceptibility of schizophrenia were found. Our findings provided new insights into the immune-inflammatory hypothesis of schizophrenia, suggesting the need for monitoring WBC count traits and the management of low-grade inflammatory responses in SCZ patients.

## Electronic supplementary material

Below is the link to the electronic supplementary material.

Supplementary Material 1: Supplementary Tables 5–16

Supplementary Material 2: Supplementary Tables 1–4, Figures 1–6

## Data Availability

The datasets supporting the conclusions of this article are included within the article and its additional files (Supplementary Information and Supplementary Tables 5–16). Publicly available summary-level datasets which were analyzed in this study could be found in Psychiatric Genomics Consortium (https://www.med.unc.edu/pgc) and GWAS Catalog (https://www.ebi.ac.uk/gwas/ home; White blood cells count: GCST90002407; Eosinophil count: GCST90002481; Basophil count: GCST90002379; Neutrophil count: GCST90002398; Monocyte count: GCST90002393; Lymphocyte count: GCST90002388). The details of the data are shown in Supplementary Table 1.

## Abbreviations

WBC: White blood cells
MR: Mendelian randomization
SNPs: Single nucleotide polymorphisms
GWAS: Genome-Wide Association Study
OR: Odds ratio
CI: Confidence interval
CRP: C-reactive protein
IVs: Instrument variables
PGC: Psychiatric Genomics Consortium
LD: Linkage disequilibrium
FDR: False discovery rates
FEP: First-episode of psychosis
