# Supplementary material for: The association between schizophrenia and white blood cells count: a bidirectional two-sample Mendelian randomization study
Source: BMC Psychiatry. 2023 Apr 19;23:271. doi: 10.1186/s12888-023-04760-6 (PMC10114369; doi:10.1186/s12888-023-04760-6)
Supplement: Supplementary file 2 — Supplementary Material 2: Supplementary Tables 1–4, Figures 1–6 [file 12888_2023_4760_MOESM2_ESM.docx]

**Supplementary Information**

**A list of Supporting Information:**

**Supplementary Table 1** Details of the datasets used in this Mendelian randomization study.

**Supplementary Table 2** The *R^2^* and *F*-statistics for the genetic instruments.

**Supplementary Table 3** The effects of Genetic predicted schizophrenia on WBC count traits in the MR analysis.

**Supplementary Table 4** The effects of Genetic predicted WBC count traits on the risk of schizophrenia in the MR analysis.

**Supplementary Fig. 1** The scatter plots of the association between genetically predicted schizophrenia on WBC count traits.

**Supplementary Fig. 2** The forest plots of the association between genetic predicted schizophrenia on WBC count traits in MR analysis.

**Supplementary Fig. 3** The funnel plots of the association between genetic predicted schizophrenia on WBC count traits in MR analysis.

**Supplementary Fig. 4** The leave-one-out analysis of the association between genetic predicted schizophrenia on WBC count traits in MR analysis.

**Supplementary Fig. 5** The scatter plots of the association between genetic predicted WBC count traits on schizophrenia in MR analysis.

**Supplementary Fig. 6** The funnel plots of the association between genetic predicted WBC count traits on schizophrenia in MR analysis.

| **Supplementary Table 1** Details of the datasets used in this Mendelian randomization study. | | | | |
| --- | --- | --- | --- | --- |
| **Factors** | **Sample size** | **Consortium** | **Source** | **Year** |
| Schizophrenia | 150064 | Psychiatric Genomics Consortium (PGC) | https://www.med.unc.edu/pgc | 2014 |
| White blood cell count | 408,112 | MRC-IEU UK Biobank | https://www.ebi.ac.uk/gwas/studies/GCST90002407 | 2020 |
| Eosinophil count | 408,112 | MRC-IEU UK Biobank | https://www.ebi.ac.uk/gwas/studies/GCST90002381 | 2020 |
| Basophil count | 408,112 | MRC-IEU UK Biobank | https://www.ebi.ac.uk/gwas/studies/GCST90002379 | 2020 |
| Neutrophil count | 408,112 | MRC-IEU UK Biobank | https://www.ebi.ac.uk/gwas/studies/GCST90002398 | 2020 |
| Monocyte count | 408,112 | MRC-IEU UK Biobank | https://www.ebi.ac.uk/gwas/studies/GCST90002393 | 2020 |
| Lymphocyte count | 408,112 | MRC-IEU UK Biobank | https://www.ebi.ac.uk/gwas/studies/GCST90002388 | 2020 |

| **Supplementary Table 2** The *R^2^* and *F*-statistics for the genetic instruments. | | | | |
| --- | --- | --- | --- | --- |
| **Exposure** | **Outcome** | **No.SNP** | ***R^2^*** | **F*F*-statistic** |
| Schizophrenia | White blood cell count | 81 | 2.16% | 31.30 |
| Schizophrenia | Basophil count | 95 | 2.57% | 37.36 |
| Schizophrenia | Eosinophil count | 85 | 2.25% | 32.69 |
| Schizophrenia | Monocyte count | 87 | 2.22% | 33.63 |
| Schizophrenia | Lymphocyte count | 76 | 2.00% | 28.86 |
| Schizophrenia | Neutrophil count | 83 | 2.32% | 32.06 |
| White blood cell count | Schizophrenia | 458 | 12.22% | 113.72 |
| Basophil count | Schizophrenia | 206 | 4.26% | 76.19 |
| Eosinophil count | Schizophrenia | 408 | 11.88% | 126.64 |
| Monocyte count | Schizophrenia | 468 | 16.53% | 162.47 |
| Lymphocyte count | Schizophrenia | 473 | 11.86% | 109.51 |
| Neutrophil count | Schizophrenia | 390 | 9.95% | 107.04 |

**Supplementary Table 3** The effects of Genetic predicted schizophrenia on WBC count traits in the MR analysis.

| **Exposure** | **Outcome** | **No. SNP** | **Methods** | **OR (95%CI)** | ***P* values** | **Egger_intercept** | ***P*-Egger_intercept** |
| --- | --- | --- | --- | --- | --- | --- | --- |
| Schizophrenia | WBC count | 81 | MR Egger | 1.026 (0.986-1.068) | 0.627 | -0.001 | 0.635 |
|  |  |  | Weighted median | 1.015 (1.006-1.025) | 0.009 |  |  |
|  |  |  | IVW (Q=173.97, P<0.001) | 1.017 (1.008-1.026) | 7.53×10^-4^ |  |  |
| Schizophrenia | Basophil count | 95 | MR Egger | 1.036 (1.004-1.069) | 0.240 | -0.002 | 0.158 |
|  |  |  | Weighted median | 1.009 (0.999-1.019) | 0.172 |  |  |
|  |  |  | IVW (Q=150.56, P<0.001) | 1.014 (1.005-1.022) | 0.002 |  |  |
| Schizophrenia | Eosinophil count | 85 | MR Egger | 1.014 (0.976-1.053) | 0.716 | 0.001 | 0.718 |
|  |  |  | Weighted median | 1.018 (1.007-1.029) | 0.009 |  |  |
|  |  |  | IVW (Q=186.59, P<0.001) | 1.021 (1.011-1.031) | 2.77×10^-4^ |  |  |
| Schizophrenia | Monocyte count | 87 | MR Egger | 1.018 (0.981-1.057) | 0.716 | -0.000 | 0.988 |
|  |  |  | Weighted median | 1.013 (1.003-1.023) | 0.034 |  |  |
|  |  |  | IVW (Q=159.89, P<0.001) | 1.018 (1.009-1.027) | 4.60×10^-4^ |  |  |
| Schizophrenia | Lymphocyte count | 76 | MR Egger | 1.043 (1.002-1.085) | 0.240 | -0.002 | 0.281 |
|  |  |  | Weighted median | 1.015 (1.005-1.025) | 0.009 |  |  |
|  |  |  | IVW (Q=146.19, P<0.001) | 1.021 (1.012-1.030) | 4.51×10^-5^ |  |  |
| Schizophrenia | Neutrophil count | 83 | MR Egger | 1.022 (0.988-1.056) | 0.627 | -0.001 | 0.616 |
|  |  |  | Weighted median | 1.015 (1.005-1.025) | 0.009 |  |  |
|  |  |  | IVW (Q=162.75, P<0.001) | 1.013 (1.005-1.022) | 0.004 |  |  |

IVW, inverse-variance weighted; WBC, white blood cell count; OR, odds ratio; CI, confidence interval

**Supplementary Table 4** The effects of Genetic predicted WBC count traits on the risk of schizophrenia in the MR analysis.

| **Exposure** | **Outcome** | **No. SNP** | **Methods** | **OR (95%CI)** | ***P* values** | **Egger_intercept** | ***P*-Egger_intercept** |
| --- | --- | --- | --- | --- | --- | --- | --- |
| WBC count | Schizophrenia | 458 | MR Egger | 0.945 (0.818-1.092) | 0.716 | 0.002 | 0.318 |
|  |  |  | Weighted median | 1.017 (0.932-1.110) | 0.845 |  |  |
|  |  |  | IVW (Q=773.35, P<0.001) | 1.009 (0.943-1.079) | 0.910 |  |  |
| Basophil count | Schizophrenia | 206 | MR Egger | 1.074 (0.876-1.318) | 0.716 | -0.002 | 0.397 |
|  |  |  | Weighted median | 1.057 (0.929-1.202) | 0.597 |  |  |
|  |  |  | IVW (Q=357.50, P<0.001) | 0.994 (0.900-1.098) | 0.910 |  |  |
| Eosinophil count | Schizophrenia | 408 | MR Egger | 0.971 (0.864-1.091) | 0.735 | 0.000 | 0.967 |
|  |  |  | Weighted median | 0.973 (0.894-1.060) | 0.711 |  |  |
|  |  |  | IVW (Q=628.66, P<0.001) | 0.973 (0.916-1.033) | 0.636 |  |  |
| Monocytes count | Schizophrenia | 468 | MR Egger | 1.028 (0.942-1.122) | 0.716 | -0.001 | 0.257 |
|  |  |  | Weighted median | 1.004 (0.933-1.081) | 0.913 |  |  |
|  |  |  | IVW (Q=771.43, P<0.001) | 0.987 (0.937-1.040) | 0.910 |  |  |
| Lymphocyte count | Schizophrenia | 473 | MR Egger | 0.969 (0.837-1.122) | 0.735 | 0.001 | 0.730 |
|  |  |  | Weighted median | 1.013 (0.922-1.113) | 0.855 |  |  |
|  |  |  | IVW (Q=867.34, P<0.001) | 0.991 (0.927-1.061) | 0.910 |  |  |
| Neutrophil count | Schizophrenia | 390 | MR Egger | 1.012 (0.868-1.180) | 0.876 | -0.000 | 0.930 |
|  |  |  | Weighted median | 1.052 (0.955-1.158) | 0.523 |  |  |
|  |  |  | IVW (Q=691.83, P<0.001) | 1.006 (0.935-1.083) | 0.910 |  |  |

IVW, inverse-variance weighted; WBC, white blood cell count; OR, odds ratio; CI, confidence interval.

**Supplementary Fig. 1** The scatter plots of the association between genetically predicted schizophrenia on WBC count traits.

a, schizophrenia on WBC; b, schizophrenia on basophil count; c, schizophrenia on eosinophil count; d, schizophrenia on monocyte count; e, schizophrenia on lymphocyte count; f, schizophrenia on neutrophil count.

**Supplementary Fig. 2** The forest plots of the association between genetic predicted schizophrenia on WBC count traits in MR analysis.

a, schizophrenia on WBC; b, schizophrenia on basophil count; c, schizophrenia on eosinophil count; d, schizophrenia on monocyte count; e, schizophrenia on lymphocyte count; f, schizophrenia on neutrophil count.

**Supplementary Fig. 3** The funnel plots of the association between genetic predicted schizophrenia on WBC count traits in MR analysis.

a, schizophrenia on WBC; b, schizophrenia on basophil count; c, schizophrenia on eosinophil count; d, schizophrenia on monocyte count; e, schizophrenia on lymphocyte count; f, schizophrenia on neutrophil count.

**Supplementary Fig. 4** The leave-one-out analysis of the association between genetic predicted schizophrenia on WBC count traits in MR analysis.

a, schizophrenia on WBC; b, schizophrenia on basophil count; c, schizophrenia on eosinophil count; d, schizophrenia on monocyte count; e, schizophrenia on lymphocyte count; f, schizophrenia on neutrophil count.

**Supplementary Fig. 5** The scatter plots of the association between genetic predicted WBC count traits on schizophrenia in MR analysis.

a, WBC on schizophrenia; b, basophil count on schizophrenia; c, eosinophil count on schizophrenia; d, monocyte count on schizophrenia; e, lymphocyte count on schizophrenia; f, neutrophil count on schizophrenia.

**Supplementary Fig. 6** The funnel plots of the association between genetic predicted WBC count traits on schizophrenia in MR analysis.

a, WBC on schizophrenia; b, basophil count on schizophrenia; c, eosinophil count on schizophrenia; d, monocyte count on schizophrenia; e, lymphocyte count on schizophrenia; f, neutrophil count on schizophrenia.
